# Energy security-related risks and the quest to attain USA’s net-zero emissions targets by 2050: a dynamic ARDL simulations modeling approach

**DOI:** 10.1007/s11356-024-32124-4

**Published:** 2024-02-13

**Authors:** Ojonugwa Usman, Oktay Ozkan, Andrew Adewale Alola, Wafa Ghardallou

**Affiliations:** 1 Department of Economics, Istanbul Ticaret University, Istanbul, Turkey; 2 Research Center of Development Economics, Azerbaijan State University of Economics (UNEC), Baku, AZ1001 Azerbaijan; 3 Adnan Kassar School of Business, Lebanese American University, Beirut, Lebanon; 4 Department of Business Administration, Faculty of Economics and Administrative Sciences, Tokat Gaziosmanpasa University, Tokat, Turkey; 5 CREDS-Centre for Research On Digitalization and Sustainability, Inland Norway University of Applied Sciences, Innlandet, Norway; 6 Faculty of Economics, Administrative, and Social Sciences, Nisantasi University, Istanbul, Turkey; 7 Department of Accounting, College of Business Administration, Princess Nourah Bint Abdulrahman University, P.O. Box 84428, 11671 Riyadh, Saudi Arabia

**Keywords:** USA, Energy security risks, Renewable energy consumption, Green technology, Dynamic ARDL simulations

## Abstract

The Russia-Ukraine war and other similar conflicts across the globe have heightened risks to the United States of America's (USA’s) energy security. However, little is known about the severity of the effect of energy security risks on the USA’s quest to attain net-zero emissions targets by 2050. To this end, we examine the effect of energy security risks on the load capacity factor (*LCF*) in the USA. Employing a time series dataset spinning from 1970 to 2018, the results of the Dynamic Autoregressive Distributed Lag (ARDL) simulations model suggest that energy security-related risk hampers the long-term net-zero emissions targets with its effect decreasing over time until it varnishes in about 5 years time. The results also show that foreign direct investment (*FDI*) inflows, renewable energy consumption, and green technology have long- and short-run positive effects on the *LCF*. Conversely, economic expansion and urbanization impede environmental quality by lowering the *LCF* both in the long run and short run. These findings are upheld by the outcomes of the multivariate quantile-on-quantile regression. Therefore, the study advocates for the consumption of renewable energy, investment in green technologies, and *FDI* inflows to mitigate energy security-related risks and attain the net-zero emissions targets by 2050 in the USA.

## Introduction

While the United States of America (USA) has consistently renewed its commitments to mitigate the agents of climate change, the country’s ecological footprint has since exceeded its biocapacity by 140 percent (The White House 2021). Despite its improvement in mitigating total emissions of GHG by roughly 7 percent compared to the level as of the 1990s, the USA is however adjudged to have the second largest carbon dioxide (CO_2_) emissions in the world. In other words, the USA is behind the People’s Republic of China when it comes to the CO_2_ emissions in the world as pontificated by the United States Environmental Protection Agency (USEPA) (2022). Besides the country’s biocapacity deficit feature as demonstrated by Global Footprint Network (2022), a complete shift to clean and renewable energy sources remains a huge challenge in the country.

With the USA’s vast economic profile, i.e., the world’s largest economy in terms of the gross domestic product (GDP), the country’s climate actions such as the clean energy transition and energy efficiency programs communicate a pathway to a successful or realization of global carbon neutrality target by 2050. However, the deficit in the USA’s biocapacity which also reflects the disproportionate ratio of biocapacity to ecological footprint, otherwise known as the load capacity factor (*LCF*), is increasingly constituting a setback to the country’s environmental sustainability (see Alola et al. 2023a). Generally, environmental sustainability has increasingly been associated with macroeconomic, socioeconomic, geographical, and other non-economic and energy-related factors. Hence, the role of energy security uncertainty is prioritized considering the USA’s energy resource especially crude oil and natural gas profile. Although the USA is a net exporter of petroleum products with net exports of 0.06 million barrels per day (b/day) as of 2021 (United States Energy Information Administration., 2022), disruptions in the global supply of energy resulting from the Russia-Ukraine war and other similar conflicts around the globe have continued to pose significant risks to the US energy risk profile.

Given that the drivers of *LCF* in the USA have not been thoroughly examined, the current study undertakes this task as an objective, especially looking at whether risks to energy security and green technology exert upward or downward pressure on *LCF* in the USA. In this direction, if increases in energy security and green technology increase the ratio of biocapacity to ecological footprint, then it suggests that the USA is well-positioned to continue improving its environmental sustainability while also edging toward its carbon neutrality target. In addition, the nature and patterns of the impact of renewable energy, *FDI*, economic expansion, and urbanization on *LCF* are also examined. While only a few studies have implemented *LCF* as an environmental indicator for the USA (Pata 2021; Pata et al. 2023b; Alola et al. 2023a), the current study expands the literature by accommodating more potential and key indicators with the use of recently developed dynamic simulated autoregressive distributed lag method (DSARDL). Specifically, our study contributes to the literature by applying the *LCF* which measures both the demand and supply aspects of the environment. Second, we use the energy security risk index of the Global Energy Institute which is based on the weights of 37 metrics, nine categories, and 4 broad sub-indices. This index captures all aspects of energy security risks, and therefore, it is adjudged to provide robust outcomes that will influence effective environmental policymaking. Third, we incorporate other main determinants of load capacity factors such as renewable energy consumption, green technology, urbanization, and economic expansion to avoid omitted variable bias problems. Fourth, we apply a dynamic ARDL simulations modeling approach with a battery of robustness checks using multivariate quantile-on-quantile regression and multivariate quantile regression. Therefore, with these contributions, it is expected that our results would perhaps provide a policy guide for the energy and environmental actors in the USA.

Therefore, our study has been obviously structured as follows: we review the related literature in the “Literature review” section. While the data and empirical approaches employed for the study are outlined in the “Data outline and model development” section, the empirical results are carefully adroitly presented and discussed in the “Empirical results” section. In the last part, i.e., “Concluding remarks and policy recommendations” section, a summary of the study alongside the policy deductions is presented.

## Literature review

### Theoretical development

The theoretical underpinning of environmental quality is rooted in the environmental Kuznets curve (EKC) hypothesis by Grossman and Krueger (1991). This preposition of the hypothesis is that stimulating income level through effective growth strategies engenders debasement of the environment at the early stage of development. This continues until income per capita attains a certain threshold after which any surge in income level apparently turns out to support the environmental sustainability. Furthermore, within the framework of the EKC hypothesis, the United Nations Environmental Protection Programme (UNEP) introduced the hypothesis of sustainable finance in 2014 as a way to mitigate the environmental consequences of growth. The main argument of this hypothesis is that to accelerate the pace of sustainable development, investment decisions are not only vital but also need to be focused on three key areas, i.e., environment, social, and governance (ESG). In this direction, United Nations Environmental Protection (UNEP (2014) emphasizes that sufficient investments in environmental protection are needed to promote clean energy transition, energy efficiency, savings, and technologies. Also, investments in the social aspect of society are encouraged to bridge income inequality and give a sense of belonging to the people thereby enhancing their productivity. Lastly, investments in governance institutions by strengthening the law and order as related to envrionmental sustainability and other aspects of development. These three factors are therefore associated with economic development in society.

Given the apparent impact of human activities on environmental externalities as documented in the early studies (Dietz & Rosa 1994; York et al. 2003; Ahmad et al. 2023a, b), there have been several modifications to the theoretical framework based on the EKC. For example, Dietz and Rosa (1994) introduced what is popularly known as the STIRPAT (stochastic impacts by regression on population, affluence, and technology). In this research, given the increasing levels of the US energy security uncertainty arising from global uncertainties such as the recent Russia-Ukraine war and other similar conflicts around the globe, this study incorporates energy security risk in the *LCF* function alongside other determinants of *LCF* such as consumption of clean (renewable) energy, green technologies, and *FDI*.

### Empirical literature

A comparative investigation of Japan and the USA was conducted by Pata (2021). This study was among the earlier studies that investigated the factors affecting the environment through *LCF* in the USA. While examining the crucial role played by clean/renewable energy and health expenditure in the *LCF* of these nations over the period 1982–2016, ARDL and other empirical approaches were applied. The results showed statistically a long-run (LR) relationship sandwiched between *LCF* and the explanatory variables in the two economies. Importantly, the LR effect of clean energy utilization and expenditure on health promotes biocapacity against ecological footprint, especially in the USA, i.e., increase in *LCF*, thus affirming the environmental desirability of the indicators. Meanwhile, the result further reveals that economic expansion via GDP damages the environment in Japan and the USA. Similarly, for the USA, Pata et al. (2023a, b, c) examined how biomass energy utilization influences *LCF* while controlling for the effects of finance and GDP per capita during the 1965–2018 period. Based on the Fourier ARDL method, their results unveiled that biomass energy increases the *LCF* and hence promotes a sustainable environment. While GDP per capita lowers the level of *LCF* (an indication of a setback to a sustainable environment), the way financial development lowers *LCF* is apparently significant only in the LR.

Furthermore, using ecological footprint as an environmental indicator, Usman et al. (2020a) examined the environmental effect of renewable (clean) energy, trade policy, biocapacity, and economic expansion between 1985 and 2014 in the USA. By implementing the ARDL and the Granger causality within the procedure outlined by Toda and Yamamoto (1995), the result showed that renewable (clean) energy and trade policy are desirable agents driving a sustainable environment in the country because they can mitigate ecological footprint. Meanwhile, the impact of biocapacity and GDP is detrimental to the environment given that the indicators both cause a surge in ecological footprint. The environmental impacts of biomass, fossil energy sources, and economic growth were also examined over the period 1981Q1–2019Q4 especially for the US transportation sector by Umar et al. (2021). The study implemented carbon dioxide (CO_2_) emissions as the environmental indicator alongside several coefficient estimation approaches and the causality approach by Breitung and Candelon (2006). The results revealed that biomass and GDP mitigate CO_2_ emissions in the country’s transport sector but fossil fuels exacerbate carbon emissions. However, in the LR, there are emissions of CO_2_ in the transport sector at different frequencies due to the increase in biomass energy utilization, fossil fuel, and economic growth.

Meanwhile, the environmental impact of green technology and related aspects has been sparsely covered in the literature (Alola & Ozturk 2021; Usman et al. 2021; Xin et al. 2021; Usman 2022, 2023). For instance, Xin et al. (2021) scrutinized whether there is an asymmetrical environmental impact of environmental-related technology in the USA. The result showed that in the economic expansion phase, positive shocks in environmental-related technology are capable of promoting a sustainable environment considering that carbon emissions decline during the period. Contrarily, negative shocks in environmental-related technology during the economic contraction phase are environmentally hazardous because an increase in carbon emissions is associated with this period. Additionally, while renewable energy utilization mitigates carbon emissions during the examined period, GDP and openness to trade activities exacerbate carbon emissions. Similarly, while affirming the validity of the EKC assumption with the use of ecological footprint as an environmental indicator, Usman et al. (2021) found that clean energy dampens carbon emission but fossil fuel energy escalates carbon emission in the USA. Meanwhile, by employing a data set that covers the period 1984–2017 for the USA, Alola and Ozturk (2021) used the ARDL approach and validated the EKC assumption. Importantly, the study revealed that high investment risks degenerate emissions while renewable (clean) energy production lessens carbon emissions. Also, using *LCF* and CO_2_ emissions as a measure of environmental indicators, Dai et al. (2023) provide support for the LCC hypothesis in the ASEAN region.

Concerning the relationship between *REC* and environmental sustainability, several studies provide evidence of the environmental improvement effect of renewable energy consumption. For example, in recent times, studies like Balcilar et al. (2023a, b) and Usman (2022, 2023) provide that as society transitions toward renewable energy, environmental improvement is bound to occur by reducing the level of emissions. In the case of the USA, Usman et al. (2020b) submit that renewable energy transitioning has a positive role in dampening emissions in the long and short terms for the USA. Similarly, Ike et al. (2020) show that the effect of renewable energy consumption is not only negative on emissions but also disparate across G7 nations. Furthermore, Iorember et al. (2022) examined the role of renewable and non-renewable energy on the environmental status of OPEC member countries in Africa. The results revealed that while renewable energy improves environmental sustainability, the effect of non-renewable energy is environmentally unfriendly.

Beyond the above-reviewed related studies, the literature on carbon neutrality or environmental sustainability has widely been extended to the role of non-energy risk or uncertainty factors (see Alola & Ozturk 2021; Syed et al. 2022; Xue et al. 2022; Ahmad et al. 2023a). Even though these studies are an extension of the literature on traditional drivers of environmental quality such as energy, economic growth, and population, they failed to capture some specific roles of energy-related risk. Thus, our study intends to fill this glaring gap using a dynamic ARDL simulations approach with a battery of robustness checks based on multivariate quantile-on-quantile regression, and multivariate quantile regression.

## Data outline and model development

### Data outline

This paper uses the US annual time series data available from 1970 to 2018 for the empirical analysis. Environmental degradation is the endogenous variable. By following a study by Alola et al. (2023a) we measure environmental degradation with the *LCF*, which considerably compares the existing biocapacity with ecological footprint. By this measurement, we demonstrate a certain ecological threshold, which means that its increases correspond to a decrease in environmental degradation and vice versa. This research includes a set of explanatory and control variables. Specifically, the explanatory variables are energy security risks, renewable/clean energy consumption, and green technology, while the control variables are economic expansion, *FDI*, and urbanization. The details of the dependent, explanatory, and control variables are given in Table 1.

**Table 1:** Details of the variables

Variable name	Symbol	Measurement	Source
Load capacity factor	*LCF*	$${\text{Biocapacity}}/\text{ecological footprint}$$(gha)	GFN Database
Energy security risk	*ESR*	Energy security risk index	GEI Database
Renewable/clean energy consumption	*REC*	Per capita energy consumption from renewables (kWh)	OWD Database
Green technology	*GTI*	Patents in environment-related technologies (% of total)	OECD Database
Economic expansion	*EG*	Per capita gross domestic product (constant 2015 US$)	WDI Database
Direct investment by foreigners	*FDI*	Foreign direct investment, net inflows (percentage of GDP)	WDI Database
Urbanization	*URB*	Population of the urban areas (percentage of total population)	WDI Database

Authors’ computation

**Fig. 1:**
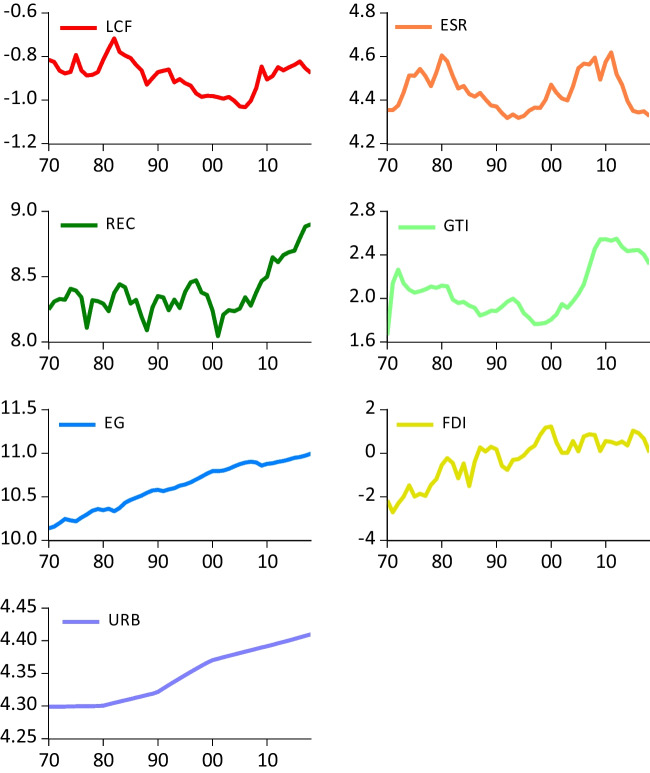
Log values of all variables spanning 1970 to 2018

Furthermore, as shown in Hassan et al. (2022), we transform all variables into logarithmic series in order to enhance homoscedasticity. Further, the logarithmic series are plotted in Fig. 1 while the statistical summary of variables is given in Table 2. Both Fig. 1 and Table 2 demonstrate that *FDI* (urbanization) fluctuates in a wider (narrower) band and also exhibits more (less) volatility compared to other variables during the sample period. As demonstrated, while the average of the annual logarithmic values of the *LCF* and *FDI* exhibit negative values, those of the other variables exhibit positive values.

**Table 2:** Variance inflation factor (VIF) or multicollinearity test

Variable	VIF	1/VIF
*EG*	9.109	0.109
*URB*	8.533	0.117
*FDI*	7.782	0.128
*GTI*	3.772	0.265
*REC*	2.794	0.358
*ESR*	1.922	0.520

Authors’ computation

Table 2 displays the test for multicollinearity based on the variance inflation factor (VIF). The results of this test suggest that the value of the VIF for each of the variables captured in this study is less than 10. This implies that there is no evidence of multicollinearity in all the variables employed in this study.

### Development of the empirical model

By and large, theoretical and empirical evidence has clearly demonstrated the possible channels through which energy security risks can affect the quality of the environment. For instance, geopolitical risk can distort the energy supply. This is more understood with the current Russia-Ukraine war and other similar conflicts across the globe, which have exacerbated the level of risks to the US energy security thereby signaling environmental deterioration and dampening economic growth. Also, the influence of clean/renewable energy in promoting a sustainable environment is well established in the literature. This is because an increase in renewable energy has little or no environmental consequences. Similarly, spending on green technology mitigates the concentration of CO_2_ emissions in the atmosphere and hence promotes the consumption of clean energy. However, the influence of economic expansion, *FDI*, and urbanization are presumed to exert negative pressure on a sustainable environment by raising the level of CO_2_ emissions.

Based on the theoretical discussion, to investigate how energy security risk, renewable/clean energy consumption, and green technology influence the USA’s *LCF* while controlling economic expansion, *FDI*, and urban population, we propose the following model:

1 $${(LCF)}_{t}={\Omega }_{0}+{\Omega }_{1}{(ESR)}_{t}+{\Omega }_{2}{(REC)}_{t}+{\Omega }_{3}{(GTI)}_{t}+{\Omega }_{4}{(EG)}_{t}+{\Omega }_{5}{(FDI)}_{t}+{\Omega }_{6}{(URB)}_{t}+{\mathbb{e}}_{t}$$

where $t$ indicates the time, $LCF$ represents the natural logarithm load capacity factor, $ESR$ is the natural logarithm of the energy security risk, $REC$ is the natural logarithm of renewable/clean energy consumption, $GTI$ is the natural logarithm of green technology, $EG$ is natural logarithm of the economic expansion, $FDI$ is the natural logarithm of direct investment by foreigners, and $URB$ is representing the natural logarithm of urbanization. Furthermore, from the model, ${\Omega }_{0}$ stands for constant term; ${\Omega }_{1}$, ${\Omega }_{2}$, and ${\Omega }_{3}$ are the slope coefficients of the factor variables; ${\Omega }_{4}$, ${\Omega }_{5}$, and ${\Omega }_{6}$ denote the slope coefficients of control variables; and ${\mathbb{e}}_{t}$ is perhaps the unobserved factors which have zero mean.

In the sense of empirical evidence, the ARDL estimation technique championed by Pesaran et al. (2001) has been widely employed in sustainable environmental studies (see Alola et al. 2019; Usman et al. 2020a, b). However, Jordan and Philips (2018) recently put up an argument that it is difficult to understand how the dependent variable responds to fundamentals in a complex model characterized by a relatively large number of lags. To overcome this deficiency, they proposed a dynamic simulated form of ARDL. This novel method first estimates the SR and LR coefficients of the model by performing stochastic simulations on the model. Then, while all other explanatory variables are fixed, the dynamic simulated ARDL model estimates the reaction of the dependent variable to counterfactual positive and negative shocks in each fundamental variable and visualizes the responses of the dependent variable automatically with impulse-response plots so that the short-run (SR) and LR impacts of the explanatory variables on the dependent variable can be clearly and easily understood (Jordan & Philips 2018).

Obviously, the dynamic ARDL has gained currency in recent studies, particularly studies that have to do with environmental sustainability (see, e.g., Agboola et al. 2022; Olasehinde-Williams and Özkan 2022; Usman 2022). This is because of its numerous advantages over the traditional ARDL model. Therefore, in the study, we develop the following error correction model based on the model in Eq. (1) and implement the dynamic ARDL simulations:

2 $${\Delta (LCF)}_{t}={\Omega }_{0}+{\theta }_{0}{(LCF)}_{t-1}+{\mathbb{s}}_{1}\Delta {(ESR)}_{t}+{\mathbb{l}}_{1}{(ESR)}_{t-1}+{\mathbb{s}}_{2}\Delta {(REC)}_{t}+{\mathbb{l}}_{2}{(REC)}_{t-1}+{\mathbb{s}}_{3}{\Delta (GTI)}_{t}+{\mathbb{l}}_{3}{(GTI)}_{t-1}+{\mathbb{s}}_{4}{\Delta (EG)}_{t}+{\mathbb{l}}_{4}{(EG)}_{t-1}+{\mathbb{s}}_{5}{\Delta (FDI)}_{t}+{\mathbb{l}}_{5}{(FDI)}_{t-1}+{\mathbb{s}}_{6}{\Delta (URB)}_{t}+{\mathbb{l}}_{6}{(URB)}_{t-1}+{\mathbb{e}}_{t}$$

**Fig. 2:**
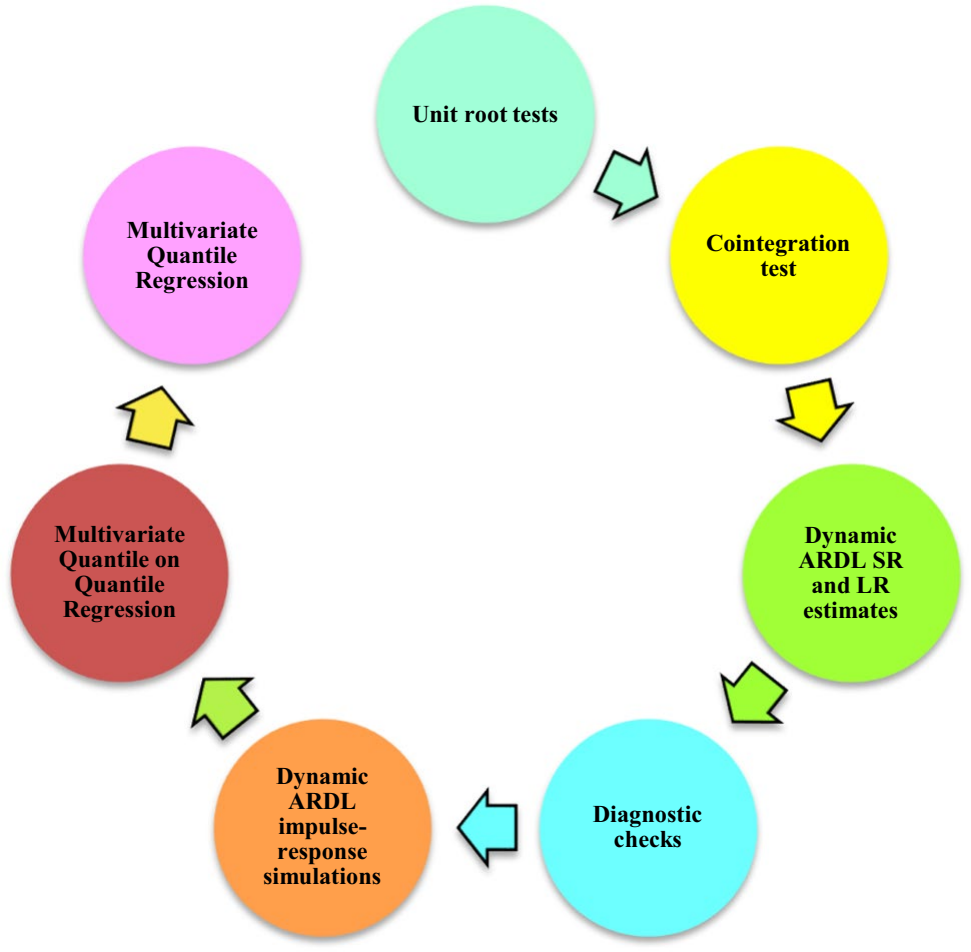
Analytical flow chart

From Eq. (2), $\Delta$ represents the first difference of each variable, ${\Omega }_{0}$ shows the estimation’s intercept, ${\theta }_{0}$ denotes the error correction term (ECT) coefficient, and ${\mathbb{e}}_{t}$ indicates the model’s error term. Additionally, ${\mathbb{s}}$ s and ${\mathbb{l}}$ s demonstrate the slope coefficients of the LR and SR impacts, respectively. The required steps for implementing the dynamic ARDL simulations model and robustness analyses are visualized in Fig. 2.

## Empirical results

### Preliminary results

The empirical analysis begins by examining the statistical descriptions of the study’s variables as shown in Table 3. The observation for each variable is 49 with the mean score of *EG* having the largest value of 10.626, followed by *REC* with 8.381 while *FDI* has the smallest value (in absolute terms) of 0.044. The description of the variables’ data also suggests that the standard deviation for each variable is less than 1 except for *FDI* which is 1.031. This, however, means that all the variables are less volatile.

**Table 3:** Summary statistics

	$LCF$	*ESR*	*REC*	*GTI*	*EG*	*FDI*	*URB*
Observations	49	49	49	49	49	49	49
Mean	− 0.888	4.441	8.381	2.083	10.615	− 0.253	4.345
Median	− 0.872	4.432	8.340	2.039	10.626	0.044	4.342
Maximum	− 0.716	4.619	8.900	2.547	10.995	1.225	4.409
Minimum	− 1.032	4.317	8.044	1.662	10.138	− 2.716	4.298
Standard Deviation	0.073	0.088	0.187	0.244	0.262	1.031	0.039

Authors’ computation

Furthermore, the next step is to probe the stationarity status of the study’s variables. This is crucial because having this information regarding the model’s variables helps to assess whether these variables are appropriate for the dynamic simulated ARDL or not; that is, the endogenous variable has to exhibit an order of integration of 1 (i.e., first difference), and that of exogenous variables not exceeding 2 as unmistakably demonstrated by Ali et al. (2022). In this regard, the Augmented Dickey-Fuller (ADF) by Dickey and Fuller (1979), Ng-Perron (NP) by Ng and Perron (2001), and Zivot-Andrews (ZA) by Zivot and Andrews (1992) are non-stationarity tests, which we remarkably apply to obtain robust results. This is similar to the approach explored in Olasehinde-Williams et al. (2021) and Özkan et al. (2022). The ADF, NP, and ZA test outcomes in Table 4 categorically demonstrate that the integration order of our endogenous variable (i.e., *LCF*) is not only 1 but also none of the explanatory variables is *I*(2). This finding implies that our variables are suitable and appropriate for the dynamic ARDL application.

**Table 4:** Outcomes of non-stationary tests

	ADF (L)	ADF ($\Delta$)	NP (L)	NP ($\Delta$)	ZA (L)	BD	ZA ($\Delta$)	BD
*LCF*	− 1.878	− 6.238^✰✰✰^	− 1.506	− 3.407^✰✰✰^	− 3.567	2008	− 6.865^✰✰✰^	1983
*ESR*	− 1.743	− 5.511^✰✰✰^	− 1.482	− 3.357^✰✰✰^	− 3.269	1999	− 6.159^✰✰✰^	1995
*REC*	− 0.721	− 7.409^✰✰✰^	− 0.576	− 3.420^✰✰✰^	− 3.340	2009	− 8.054^✰✰✰^	2002
*GTI*	− 1.423	− 7.410^✰✰✰^	− 0.532	− 0.937	− 4.850^✰^	2004	− 8.704^✰✰✰^	2010
*EG*	− 1.537	− 5.135^✰✰✰^	0.968	− 3.304^✰✰✰^	− 4.759^✰^	2009	− 5.551^✰✰✰^	1983
*FDI*	− 2.183	− 7.906^✰✰✰^	− 1.075	− 3.338^✰✰✰^	− 4.021	1978	− 6.119^✰✰✰^	1997
*URB*	− 1.010	− 1.949	− 4.999^✰✰✰^	− 0.902	− 7.353^✰✰✰^	1991	− 3.594	2000

^✰✰✰^ and ^✰^ denote 0.01 and 0.10 significance levels, respectively. Since the results of *MZ*_*a*_, *MZ*_*t*_, *MSB*, and *MPT* are the same, we only report the *MZ*_*t*_ outcomes of the NP unit root test. *BD* stands for break date estimated by the ZA unit root test

Since the dynamic simulated ARDL method requires a cointegration relationship, the Pesaran-Shin-Smith (PSS) bound testing method with Narayan’s (2005) critical values is obviously applied to investigate the cointegration association between the study’s variables. Essentially, the PSS bound testing results in Table 5 divulge that the absolute value of the computed *F*-statistics and *t*-statistic is higher than the critical value of the upper bound at the 1% significance level. This remarkably indicates a statistically significant cointegration association. This result symbolizes that the cointegrating requirement of the dynamic simulated ARDL application is also met.

**Table 5:** PSS bound test results

Test statistics	Value	*K*	*H*_0_	*H*_1_
*F*-statistic	6.004^✰✰✰^	6	No cointegration	Cointegration
*t*-statistic	− 5.308^✰✰✰^			
Critical values				
Significance	***F*-statistic**		***t*-statistic**	
	${\varvec{I}}$**(0)**	${\varvec{I}}$**(1)**	${\varvec{I}}$**(0)**	${\varvec{I}}$**(1)**
10%	2.31	3.51	− 2.57	− 4.04
5%	2.73	4.06	− 2.86	− 4.38
1%	3.66	5.33	− 3.43	− 4.99

$I$(0) and $I$(1) represent lower and upper bounds; ^✰✰✰^ denotes 0.01 significance level

### Results of short- and long-run relationships

After determining the variables appropriate for the dynamic simulated ARDL model, we therefore evaluate the LR and SR impacts of energy security risk, renewable/clean energy consumption, and green technology on the *LCF* while controlling economic expansion, *FDI*, and urbanization. Note that we set the number of simulations to as high as 10,000 in the dynamic ARDL application. The outcomes of the analysis are reported in Table 6. From the table, it can be seen that the coefficients of the factor variables show that the SR impact of energy security risk on *LCF* is negative and significant, whereas the impact of renewable/clean energy utilization and green technology is positive and statistically significant in the LR. Specifically, a 1% increase in energy security risk lowers *LCF* by 0.260% in the SR. On the other hand, a 1% increase in renewable/clean energy utilization and green technology stimulates the *LCF* by 0.114% and 0.078% in the LR, respectively. These results categorically reveal that energy security risk has an adverse effect on the environment in the USA, while the utilization of renewable/clean energy and green technology improves the environment toward sustainability.

**Table 6:** Dynamic simulated ARDL LR and SR estimates

Parm	Estimate	Standard error	*t*-value	*p*-value
Intercept	3.600^✰✰^	1.566	2.298	0.027
*LCF*_*t* − 1_	− 0.477^✰✰✰^	0.089	− 5.308	0.000
$\Delta$ *ESR*_*t*_	− 0.260^✰✰✰^	0.088	− 2.944	0.005
*ESR*_*t* − 1_	0.009	0.062	0.145	0.885
$\Delta$ *REC*_*t*_	0.064	0.043	1.472	0.150
*REC*_*t* − 1_	0.114^✰✰^	0.042	2.675	0.011
$\Delta$ *GTI*_*t*_	0.074	0.044	1.670	0.104
*GTI*_*t* − 1_	0.078^✰✰^	0.036	2.165	0.037
$\Delta$ *EG*_*t*_	− 0.824^✰✰✰^	0.220	− 3.746	0.000
*EG *_− 1_	− 0.076	0.100	− 0.761	0.451
$\Delta$ *FDI*_*t*_	0.013	0.009	1.437	0.159
*FDI *_− 1_	0.025^✰✰^	0.011	2.160	0.037
$\Delta$ *URB*_*t*_	− 0.813	3.610	− 0.225	0.822
*URB *_− 1_	− 1.003^✰^	0.569	− 1.760	0.087
*R*^2^	0.737		Simulations	10,000
Adj. *R*^2^	0.636		Prob > *F*	0.000

^✰✰✰^, ^✰✰✰^, and ^✰^ denote 0.01, 0/05, and 0.10 levels of significance

**Fig. 3:**
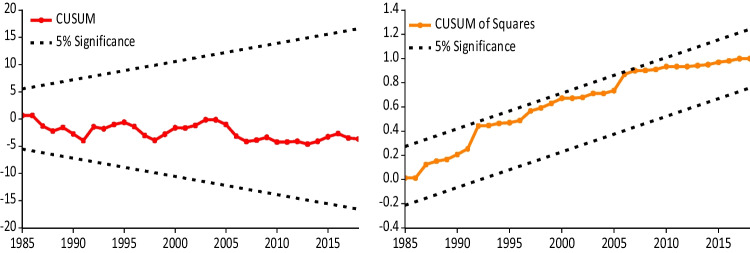
Outcomes of CUSUM and CUSUM of sq. tests

Looking at the outcomes of the control variables in the estimations, we see that dynamic growth expansion has a significant SR impact on the *LCF*, while both *FDI* and urban population have an LR impact. Empirically, a 1% surge in economic expansion causes a 0.824% decline in *LCF* in the SR. Furthermore, a 1% rise in *FDI* increases the *LCF* by 0.025%, while a 1% increase in urbanization reduces the *LCF* by 1.003% in the LR. These outcomes demonstrate that economic expansion and urbanization deteriorate the USA’s environmental quality, whereas it is positively affected by the *FDI*. Furthermore, the results in Table 7 and Fig. 3 disclose that our dynamic ARDL model given in Eq. (2) has no diagnostic issue, indicating that the obtained findings from Table 5 are reliable and the model is adequate.

**Table 7:** Diagnostic checks

Test name	Stat	*p*-value
Breusch-Godfrey LM	0.903	0.348
Breusch-Pagan-Godfrey	0.705	0.744
ARCH	0.016	0.897
Jarque–Bera	0.699	0.704
Shapiro–Wilk	0.979	0.541
Ramsey-reset	2.881	0.100

Authors’ computation

**Fig. 4:**
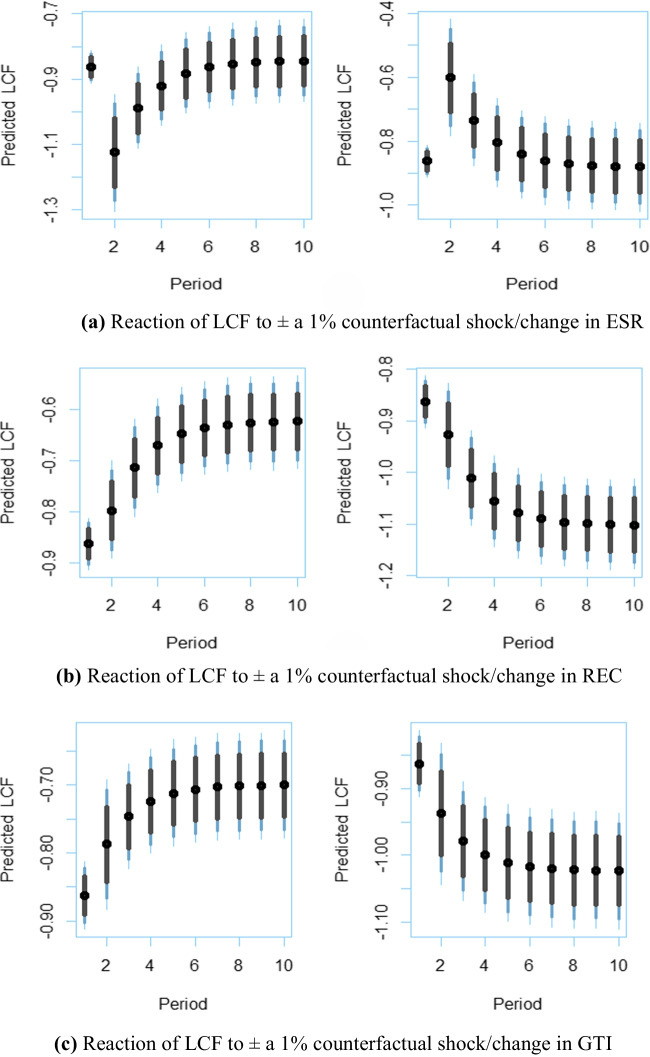

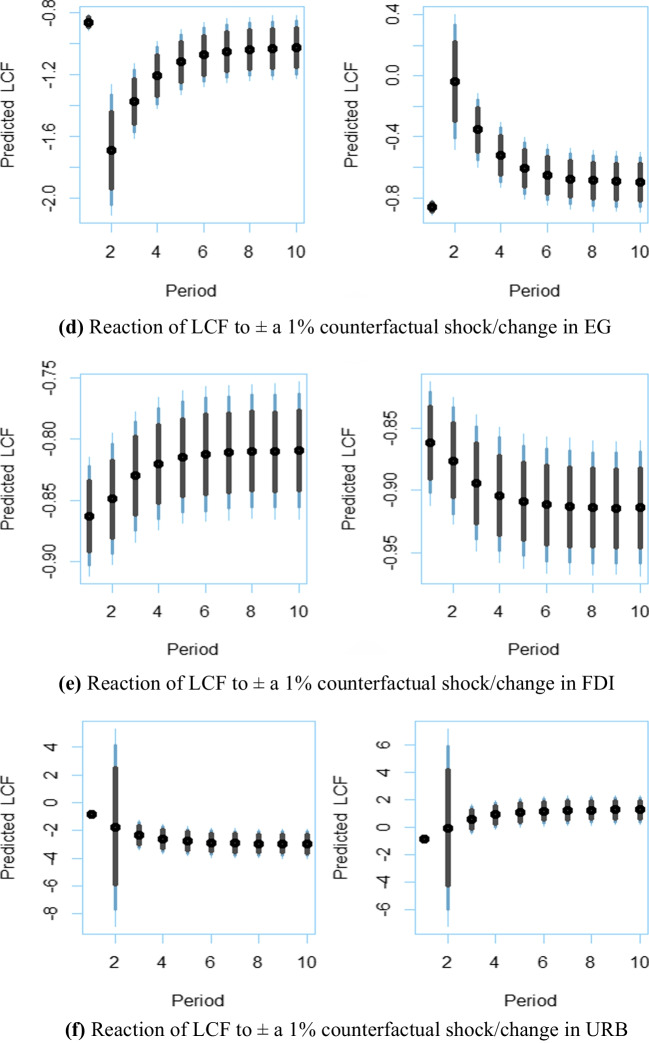
Dynamic ARDL impulse-response simulation plots. The black dots represent the predicted mean logarithmic *LCF* values for the relevant periods. The 75%, 90%, and 95% confidence intervals are represented by shaded vertical lines (from gray to light blue). The vertical lines range from gray to light blue

Finally, we investigate the SR and LR responses of the *LCF* to a 1% counterfactual positive or negative shock in exogenous variables.^1^ The impulse-response plots automatically produced by the dynamic stimulated ARDL are exhibited in Fig. 4. The plots on the left side show the responses of the *LCF* to a + 1% counterfactual shock, while those on the right side demonstrate the response to a − 1% counterfactual shock. Figure 4(a) indicates that a 1% positive (negative) change in energy security risk significantly reduces (increases) the *LCF* in the SR, but its effect on the environment decreases over time and subsequently disappears in about 5 years. On the other hand, Fig. 4(b), (c), and (e) display that a 1% positive (negative) change in renewable/clean energy utilization, green technology, and *FDI* increases (decreases) the *LCF* both in the SR and LR. Moreover, Fig. 4(d) demonstrates that a 1% positive (negative) change in economic expansion significantly decreases (increases) the *LCF* in the SR, but its negative effect on the environment declines over time. Following the Narayan and Narayan (2010) approach, the decrease in the positive effect of economic expansion over time indicates movement toward validating the EKC proposition. In this regard, since Fig. 4(d) reveals that the positive effect of economic expansion decreases over time in the USA, we conclude that the EKC proposition is likely to be validated for the USA Lastly, Fig. 4(f) shows that a 1% positive (negative) change in urbanization reduces (surges) the *LCF* both in the SR and LR; that is, urbanization negatively affects environmental sustainability plan in the USA.

### Robustness checks

**Fig. 5:**
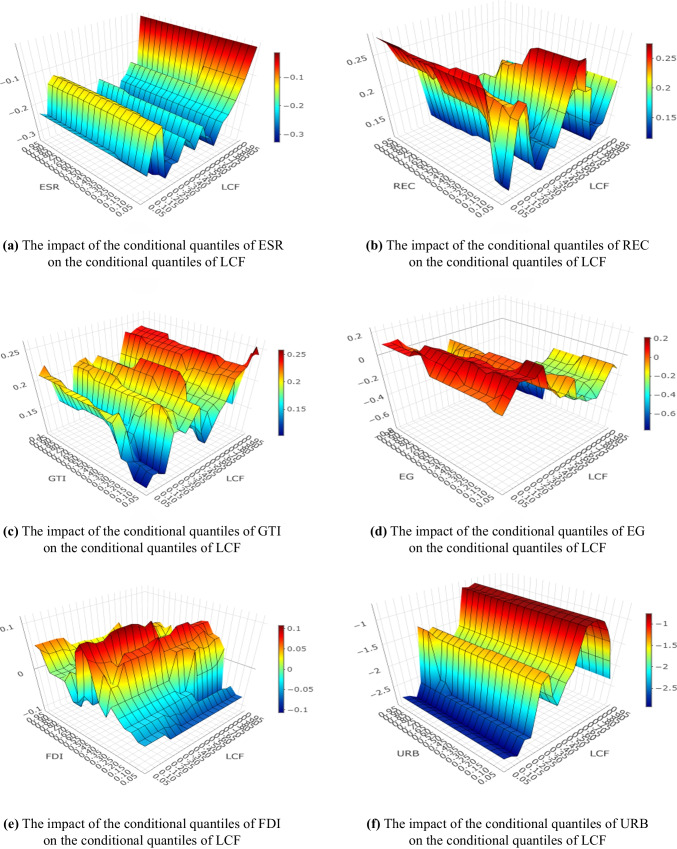
Multivariate quantile on quantile plots

The Multivariate Quantile on Quantile Regression (MQQR) approach introduced by Alola et al. (2023b) is employed to check the results of the dynamic simulated ARDL model. Figure 5 illustrates the MQQR estimates. Figure 5(a) and (f) reveal that the impact of *ESR* and *URB* on *LCF* is negative for all pairwise quantiles, respectively. On the other hand, Fig. 5(b) and (c) demonstrate that the impact of *REC* and *GTI* on *LCF* is positive for all pairwise quantiles, respectively. Furthermore, Fig. 5(d) and (e) exhibit that although the effect of *EG* (*FDI*) on *LCF* is positive (negative) for some quantiles, it is negative (positive) for the majority of quantile pairs. These findings based on the MQQR empirically support the findings of the dynamic simulated ARDL model.

**Fig. 6:**
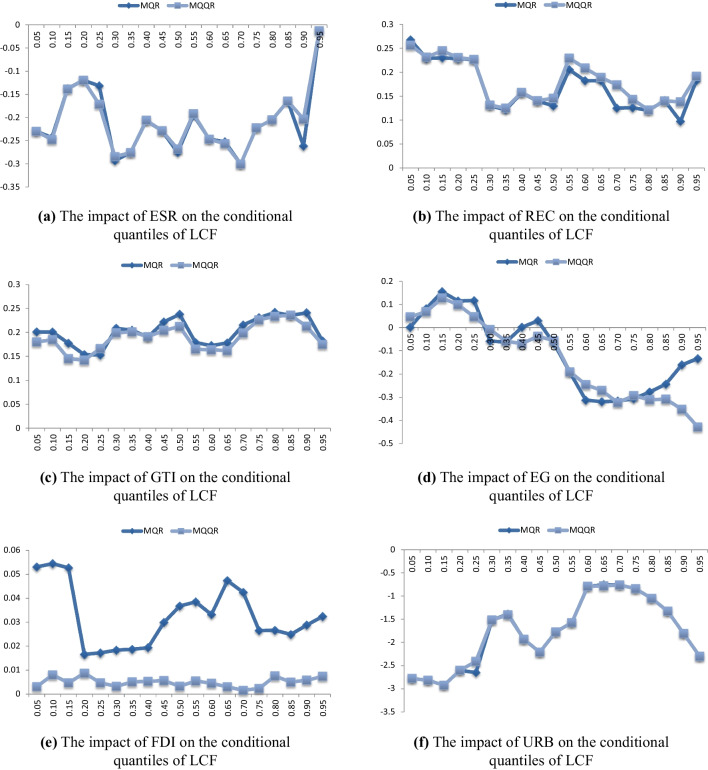
Comparison of the multivariate quantile regression and averaged multivariate quantile on quantile regression estimates

Furthermore, following the studies of Olanipekun et al. (2023) and Ozkan et al. (2023b), we employ quantile regression (QR) to check the robustness of the results of the quantile-on-quantile regression (QQR) results; we also apply the multivariate quantile regression (MQR) to check the robustness of the MQQR findings. Figure 6 demonstrates the MQR and averaged MQQR estimates. It is evident from Fig. 6(a)–(f) that the MQR and averaged MQQR estimates are quite similar, suggesting the robustness of the findings based on the MQQR.

### Discussion of main findings

The results based on the dynamic ARDL and its simulated impulse responses suggest that *ESR* lowers the decarbonization plan in the SR by reducing the degree of *LCF*. Similarly, the counterfactual shock responses of environmental improvement measure validate the results, and in addition, show that the responses of *LCF* to a shock in *ESR* decrease over time and subsequently disappear after 5 years. The implication of this finding is that an increase in the uncertainties related to energy security promotes environmental degradation by reducing the *LCF* of the USA. However, the effect of *ESR* continues to dampen across the distribution of *LCF* until it vanishes around the 5th year. This finding plausibly suggests that strong environmental policies, which have been put in place, may result in the dampening environmental effect of energy-related risks and uncertainties in the USA. Therefore, this finding echoes the major conclusion of Usman et al. (2020a) that clean energy consumption promotes environmental quality only in the LR.

Furthermore, our outcomes/findings show that the transition to renewable/clean energy utilization promotes the US environmental quality through its positive impact on the *LCF*. The underlying reasons for this outcome are that renewables are clean energy sources and as such extractions, processing, and utilization do not have carbon dioxide emission contents that disrupt the environment. Even though fossil fuels have some economic gains, the process of generating unclean fossil fuels exacerbates the accumulation of emissions, particularly CO_2_ in the atmosphere thereby deteriorating environmental quality. This finding echoes the major conclusion in Alola and Ozturk (2021) for the USA, Ike et al. (2020) for G-7 nations, Usman (2022) for Nigeria, Balcilar et al. (2023a) for 34 African nations, Özkan et al. (2023a) for India, and Özkan et al. (2023a) for USA and EU.

Moreover, the role of spending on green energy is found to stimulate environmental sustainability by increasing the ratio of biocapacity to ecological footprint. This is possibly explained by the increasing renewable energy contents in technological energy innovations. As the level of energy generated from renewables increases, the rising sea levels are reduced by lowering the concentration of CO_2_ emissions in the atmosphere. This is responsible for the heavy calls via the UN Climate Change Conferences to limit the global temperature rise to or below 1.5 °C and double the renewable/clean energy mix in their total energy utilization. Therefore, our outcomes concur with Balcilar et al. (2023b) for OECD countries, Usman et al. (2021) for the USA, Usman (2023) for G7 countries, and Ozkan et al. (2023c, d) for Turkey and China, respectively.

In furtherance of the discussion of the findings of this study, *FDI* brings the USA to a sustainable path by increasing clean technology and improving environmental standards. This signals that environmental laws and regulations are stringent and enforceable without compromise. Therefore, our finding affirms the halo effect hypothesis which submits that MNCs disseminate superior knowledge, which eventually leads to environmentally friendly practices. On the contrary, the finding of this study is in disagreement with Balcilar et al. (2023a), who found that the TNCs who undertake *FDI* behave in such a way that leads to environmental damage in African countries. Ozkan et al. (2023a) and Pata et al. (2023a) also revealed the negative impact of *FDI* on environmental quality in China. This is because the motive behind their operations is anchored on profit accumulation.

Further, the urban population tends to be negatively connected with the environment. This implies that the urban population reduces the environmental sustainability plan by reducing the amount of *LCF*. Theoretically and empirically, an increase in urban population increases the usage of energy and economic expansion. The upward pressure on the amount of energy consumed may increase the consumption of energy from the oils thereby increasing the accumulation of emissions in the atmosphere. This result agrees with Usman et al. (2021), and Pata et al. (2023c) who all found that an upsurge in environmental pollution is traceable to rapid urbanization. On the contrary, Shahbaz et al. (2016) presented that the urban population improves energy efficiency and savings thereby reducing the environmental effect of energy consumption.

**Fig. 7:**
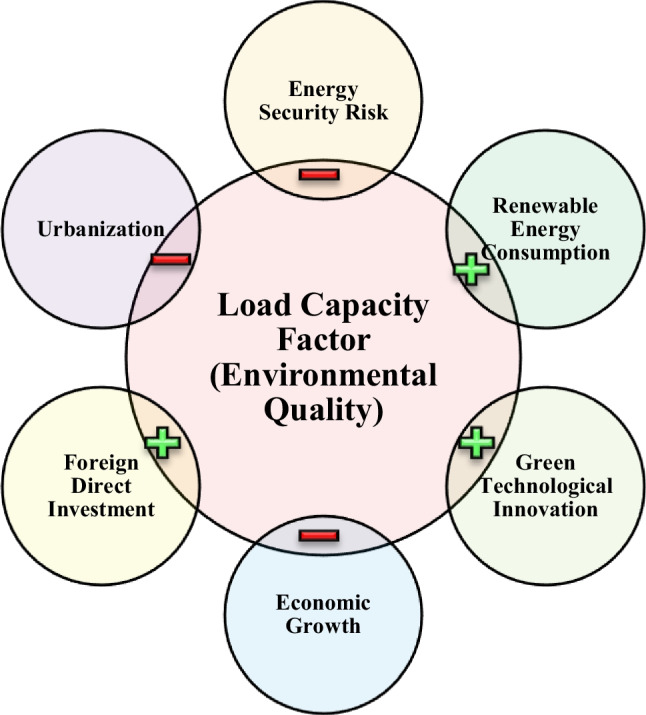
Summary of the findings

We summarize the findings of the study in Fig. 7. The figure implies that renewable/clean energy usage, green technology, and *FDI* increase the *LCF* (i.e., improve a sustainable environment), while energy security risk, economic expansion, and urban population decrease the *LCF* (i.e., deteriorate a sustainable environment).

## Concluding remarks and policy recommendations

In this study, we examine the environmental effect of energy security risks, renewable/clean, and green energy innovation through spending on R&D technologies in the USA by controlling for economic expansion, *FDI*, and urbanization. We use the dataset spanning from 1970 to 2018. To achieve this, we employ the ARDL simulations model to ascertain the SR and LR slope parameters of the fundamental variables — both core and control variables. The main empirical results show that renewable/clean utilization, green technology, and *FDI* promote the environment through a rise of the *LCF* on the one hand. On the other hand, energy security-related risks, economic growth, and urbanization deteriorate the environment by lowering the *LCF*. The positive effect of growth with *LCF* signals validating the EKC proposition. In addition to these results, the *LCF* reactions to a counterfactual shock suggest diminishing effects of *ESR* which eventually disappear over time.

### Policy recommendations

Based on these findings, the study suggests several policy recommendations that tend to aid the United Nations Sustainable Development Goal (UNSDG) 13, i.e., implementing urgent action to address climate change and its impacts. First, the negative effect of *ESR* on the environment and its subsequent disappearance suggest that to realize the USA’s ambitious net-zero emission targets by 2050, risks in energy-related resources need to be curtailed. Although energy-related emissions are diminishing probably because of the conserted efforts to push up the total clean energy in the energy mix of the country.

Second, the positive effect of renewable/clean energy utilization with the *LCF* is a signal that the USA is currently in line with the UNSDGs 7 and 9 while aiming to achieve environmental sustainability given the enormous resources available in the country. On this note, we suggest that the country should double the level of investments in clean and renewable energy to speed up the pace of energy transition toward the path of realizing the LR targets of net-zero emissions. This kind of investment is important in terms of the decarbonization of the economy. Therefore, to achieve net-zero emission levels, carbon pricing as a policy should be enforced to help stimulate investments in renewables and reduce return rates on fossil fuel energy assets. Similarly, there is a need to subsidize renewable energy projects, maybe by lowering their lending rate. This will have implications not only for energy savings but also for energy efficiency technologies.

Third, there is a need to significantly improve the expenditure on research and development, particularly as regards renewable energy technologies. Adopting better ways of producing efficient energy through green technology would accelerate government intents to substitute fossil fuels for renewable energy. Therefore, renewable energy technologies should be embraced and scaled up at industrial and household levels.

Fourth, although the behaviors of MNCs regarding their operation via *FDI* are environmentally friendly. However, since firms and corporations owned and controlled by domestic and foreign investors are set up to maximize profits, the government and policymakers should not relent in their efforts to make sure that the existing environmental laws and regulations are strictly adhered to. In other words, there should be no occasion for regulatory forbearance as any investors who violate environmental laws should be dealt with accordingly. In addition, government and policymakers should be cautioned not to over-zealously formulate environmental policies that discourage investment in the energy sector such as high environmental and carbon taxes.

Fifth, economic growth and urbanization are channels of environmental pollution in the USA. Therefore, we suggest that growth policies should have green content. In other words, growth policies should encourage the utilization of clean energy such as solar, hydrogen, wind, biomass, geothermal, and nuclear energy in order to address environmental catastrophes in the atmosphere. Additionally, urban migration should be discouraged to reduce excessive demand for energy consumption in urban areas, which leads to over-stretching of resource utilization.

Finally, like any other study, this study is faced with some limitations. The analysis of this study is based on the USA which has no similar features to developing and emerging market economies. Therefore, the results arising from this study may not be applicable in entirety for developing and emerging market economies.

## Data Availability

Load capacity factor is obtained from the Global Footprint Network database. Energy security risk index is obtained from the global energy institute https://www.globalenergyinstitute.org/energy-security-risk-index. Renewable energy consumption is collected from the database of our word in data. Foreign direct investment, urbanization, and GDP per capita are all collected from the database of the world development indicators. Patents in environment-related technologies (% of total) was correlected from the OECD database.

## Footnotes

1 1 We use the counterfactual shock of 1% based on the research conducted by Sarkodie et al. (2019, 2022), Danish and Ulucak (2020, 2022), and Shahabad and Balcilar (2022).
